# On the optimal choice of a hyperelastic model of ruptured and unruptured cerebral aneurysm

**DOI:** 10.1038/s41598-019-52229-y

**Published:** 2019-11-01

**Authors:** D. V. Parshin, A. I. Lipovka, A. S. Yunoshev, K. S. Ovsyannikov, A. V. Dubovoy, A. P. Chupakhin

**Affiliations:** 1 Lavrentyev Institute of Hydrodynamics, SB RAS, Lavrentyev av 15, Novosibirsk, 630099 Russian Federation; 2 Novosibirsk State University, Novosibirsk, 630090 Russian Federation; 3 Federal Neurosurgical Center, Novosibirsk, 630048 Russian Federation

**Keywords:** Tissues, Vascular diseases

## Abstract

In the last decade, preoperative modelling of the treatment of cerebral aneurysms is being actively developed. Fluid-structure interaction problem is a key point of a such modelling. Hence arises the question about the reasonable choice of the model of the vessel and aneurysm wall material to build the adequate model from the physical point of view. This study covers experimental investigation of 8 tissue samples of cerebral aneurysms and 1 tissue sample of a healthy cerebral artery. Results on statistical significance in ultimate stress for the classification of 2 cohorts of aneurysms: ruptured and unruptured described earlier in the literature were confirmed (*p* ≤ 0.01). We used the four most common models of hyperelastic material: Yeoh, Neo-Hookean and Mooney-Rivlin (3 and 5 parameter) models to describe the experimental data. In this study for the first time, we obtained a classification of hyperelastic models of cerebral aneurysm tissue, which allows to choose the most appropriate model for the simulation problems requirements depending on the physical interpretation of the considered problem: aneurysm status and range of deformation.

## Introduction

An intracranial aneurysm (IA) is a cerebrovascular disease, which approximately occurs in 1 out of 50 people^1,2^. Usually unruptured aneurysms are discovered accidentally, and the techniques of surgery are controversial since the risks of postoperative complications and the risk of aneurysm rupture are comparable^2,3^. One of the main concerns of the modern neurosurgery is how to assess the risk of rupture of certain aneurysm and the urgent task of preoperative monitoring is determining the tactics for treatment of aneurysm depending on the dynamics of the development of such pathology. Modern methods of pre-operational computer modelling^4–6^ can be used for this task. For that kind of simulation, both 3D models with rigid walls and fluid structure interaction (FSI) models are used. The latter allow the most accurate consideration of the characteristics of the material of the aneurysm wall in solving FSI problem, but their complexity lies in determining the characteristics of the material of the wall of the cerebral aneurysm. In this study, we analysed 8 samples of cerebral aneurysms walls, belonging to 6 women and 2 men mostly of similar age. In addition to experimental data on cerebral aneurysm, we present a similar study protocol for the patient’s healthy superficial temporal artery that was turned off from the circulation for microsurgical treatment of cerebral aneurysm. Data on the patients are given in Table 1.

**Table 1:** Patient and specimen characteristics. Only circulation or systemic diseases are under consideration, such as: H – hypertension, Hep – hepatitis, D – diabetes, S – smoking, C – cholelithiasis, IS - ischemic stroke, HS – hemorrhage type stroke, Tbc – tuberculosis, A – bronchial asthma, UD – urolithiasis disease, O – obesity, Tbp – thrombophlebitis, Paf – paroxysmal atrial fibrillation.

ID	Gender (Age)	Aneurysm location	Average thickness (*MM*)	Average width (*MM*)	Cross-sectional area (*MM*^2^)	Status	Strain limit	Stress limit (MPa)	Risk factors
R.	F (63)	MCA	0.05	3	0.15	Unruptured	1.27639	1.09936	H+Tbp+Paf
K2.	F (55)	MCA	0.05	4	0.2	Unruptured	1.5729	1.1314	IS+H
V.	F (63)	AComA	0.15	3.75	0.56	Ruptured	1.06323	1.05493	HS+UD
Z.	F (48)	MCA	0.2	3.3	0.66	Ruptured	0.744889	1.00979	HS+H
U.	F (43)	PICA(L)	0.25	3.56	0.91	Ruptured	2.85794	1.00356	HS+H+A
K1.	F (63)	MCA	0.3	5	1.5	Unruptured	4.6129	1.75217	HS+H+D+*O*_3*grade*_
M.	M (38)	AComA	0.7	4	2.8	Ruptured	1.30277	0.382825	HS+H+S+Tbc
Ul.	M (44)	AComA	0.6	3.85	2.31	Ruptured	3.96535	0.33317	HS+H+Hep+D+S+C
A1.	M (60)	TempA	0.4	3	1.2	Not applicable	1.41559	2.4794	IS
A2.	M (60)	TempA	0.2	1	0.2	Not applicable	1.37917	0.141357	IS

The purpose of our work was to study the changes in the mechanics of the material of the wall of the cerebral aneurysm in the experiment and in mathematical modelling during the increase in engineering strain of the sample in uniaxial tensile test. Since in the majority of papers authors do not justify the choice of certain hyperelastic material model for modelling mechanics of IA, we intended to answer the question: which hyperelastic model should be used for a particular FSI numerical simulation problem? Our work provides the answer to this question in the context of ruptured and unruptured aneurysms, as well as depending on the level of deformation of the aneurysm tissue.

## Materials and Methods

### Clinical protocol

All cerebral aneurysm tissue specimens were harvested during the microsurgical clipping. This part of research was carried out in cooperation with Federal Neurosurgical Centre of Novosibirsk. The obtained tissue was preserved with saline 0.9% at +2 C-+5 C during transportaton(12–48 h). According to^7^ refrigerated vessel walls, being loaded, show slightly different results from freshly obtained tissue with relation to used temperature. Similar protocol was used earlier in^8^ in the study of the rheology of cerebral aneurysms. After delivery to the laboratory, rectangular shape is cut from the specimen, and its size measured. Then the sample was fastened in tensile machine (Zwick&Roell Z10, Germany) and a series of experiments was performed.

The part of work was performed in accordance with the ethical committee: “Local ethical committee of the Federal Neurosurgical Center (Novosibirsk)”. According to the legislation of the Russian Federation, the material of pathological tissues becomes class B medical waste immediately after separation from the patient, according to the policy of the ethical committee, studies conducted using such material require either anonymization of patient data or their informed consent. In this work, patient data is anonymized, since the surgeon determines the possibility and necessity of extracting the tissue of cerebral aneurysm only directly during the operation. All experimental techniques were approved by “Local ethical committee of the Federal Neurosurgical Center (Novosibirsk)”.

### Experimental setup protocol

Experiments aimed to determine the mechanical properties of the walls of cerebral aneurysms were first initiated in^9^. At present, there are several experimental approaches. The most common experimental approach is uniaxial loading on a tensile machine. A similar approach was used in such works as^8,10,11^ and others. This technique allows to locate the area of deformation of the biomaterial rather well. However, there are approaches with multiaxial loading^12,13^. The main difficulty in such approaches is the definition of the zone of two-dimensional deformation and a significant dependence on fixing the sample in the machine^14^. Even in the case when the technique of hooks is chosen, there are specific features of the propagation of a discontinuity in the tissues, which essentially depends on the fixing of the material correctly, and not on its own properties^15^. We selected the technique described in^8^ with applying the sandpaper to the clamps.

An uniaxial tensile machine Zwick&Roell Z10 was used for mechanical testing of the samples. The special feature of this machine, unlike in the works^10,16^ was the vertical setup of the tensile test which minimises the influence of friction on the measurement result. In the work^16^ zone of local deformations is observed visually, by measuring the distance between the punctures in the tissue. In the^10,17^ the deformation is determined by change in the location of the traverse – we used the same approach. Despite the fact that the first approach is more correct in the sense of taking into account the boundary conditions for fixation of the material, the second is also actively used in the experimental practice. This is due to the fact that set of parameters differs from work to work: storage conditions, model of a tensile machine, the environment of the experiment, the number of cycles used for preconditioning. Taking into account the high level of distinction between the characteristics of the tissue of aneurysms, the comparison of the strength properties of the walls of aneurysms obtained in our work is within the limits of the deviations described in^11^.

**Figure 1:**
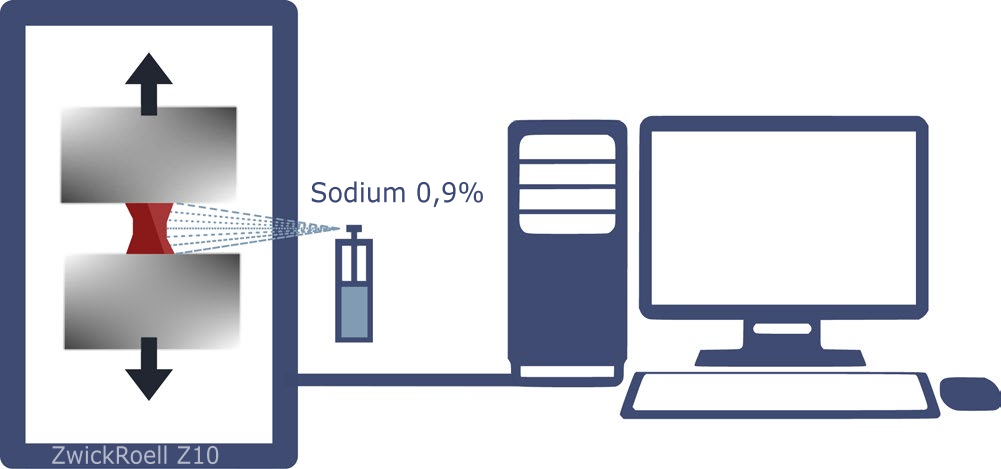
The experimental setup.

For each sample we performed a series of experiments each of those was divided into several stages. The number of steps varied from sample to sample, depending on the size of the current specimen (for very thin samples, only one step was possible, for extremely thick and strong samples – up to 10 stages). While carrying out the experiment, we took into account such a well-known phenomenon for biological tissues, as preconditioning^18^. The need to take into account this phenomenon was due to the complex fibrous microstructure of the aneurysm wall^8,19^. Significant role in the mechanics of such tissues is played by the matrix of a similar fibrous sample. Considering the relaxation of the matrix during the experiments ensures that the true stresses in the sample are correctly taken into account. We applied this technique for the initial stages (1th–5th stage for each elongation step depending on the sample), and during next stages the influence of this condition was not noticed. During our study we established that there’s no need to perform more than two preconditioning cycles. For each stage of the experiment for certain aneurysm specimen, its initial elongation was the same: i.e. after the completion of each stage of the loading, the machine’s clamps returned to the original, program defined position. After every stage of experiment the sample was moistened with physiological saline (Fig. 1). Since the aneurysm tissue was quickly degenerating under loading and becoming unfit for the further study, it was possible to carry out only one series of experiments for each sample. Speed of pulling was equal 1 mm per minute and was the same for all experiments. In each experiment, the sample lost its elasticity. For each sample, its loading was performed until it was separated into two disconnected segments (or a visible discontinuity of the sample appeared).

### Mathematical models of hyperelastic material of intracranial aneurysm

As many other live tissues, aneurysm samples were considered an hyperelastic material^10,17,20^. The data obtained during the tensile tests were used to construct mathematical models of the material of the cerebral aneurysm wall. We considered two Mooney-Rivlin models (3 and 5 parameter), Yeoh model and Neo-Hookean model. Computing the parameters for the models was performed with Wolfram Mathematica 11.3, Licence of LIH SB RAS. Using built-in *Fit* program function based on least squares method, the initial approximation was obtained, that served the base for approximation with a model. To construct the approximation we used the part of the curve up to the plateau zone, after which the zone of plasticity starts, and using the hyperelastic model for its modelling is inappropriate.

**Figure 2:**
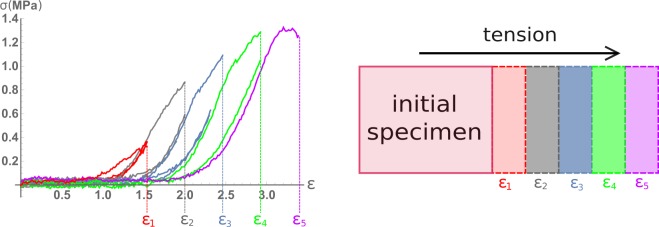
Illustration of the idea of research with increasing engineering strain of intracranial aneurysms specimen. Each engineering strain shown in the figure and numerically expressed in Table 2 corresponds to the lifespan of the aneurysm, as it would have continued to grow inside the patient.

Gathered experimental data were analysed and processed, followed by plotting graphs for every specimen. Different stages were colour-marked, with colours corresponding to number of the stage (Fig. 2).

The Mooney-Rivlin model is a special case of the generalised Rivlin model:

1 $$W=\mathop{\sum }\limits_{p,q=0}^{N}{C}_{pq}{({\bar{I}}_{1}-3)}^{p}{({\bar{I}}_{2}-3)}^{q}+\mathop{\sum }\limits_{m=1}^{M}{D}_{m}{(J-1)}^{2m}$$

Where *W* – strain energy density function, *C*_*pq*_ – material constants related to the distortional response, *D*_*m*_ – material constants related to the volumetric response. As the conducted tension of aneurysm tissue in mechanic tests was uniaxial the mathematical models will be considered under that condition. Assuming the studied hyperelastic material is incompressible, this representation of Cauchy stress follows:

2 $${\sigma }_{3p}=\frac{F}{{S}_{0}}=2{C}_{1}(\lambda -\frac{1}{\lambda })+2{C}_{2}(1-\frac{1}{{\lambda }^{3}})+6{C}_{3}({\lambda }^{2}-\lambda -1+\frac{1}{{\lambda }^{2}}+\frac{1}{{\lambda }^{3}}-\frac{1}{{\lambda }^{4}}),$$

3 $$\begin{array}{rcl}{\sigma }_{5p}=\frac{F}{{S}_{0}} & = & 2{C}_{1}(\lambda -\frac{1}{\lambda })+2{C}_{2}(1-\frac{1}{{\lambda }^{3}})+6{C}_{3}({\lambda }^{2}-\lambda -1+\frac{1}{{\lambda }^{2}}+\frac{1}{{\lambda }^{3}}-\frac{1}{{\lambda }^{4}})+\\  &  & +4{C}_{4}\lambda (1-\frac{1}{{\lambda }^{3}})({\lambda }^{2}+\frac{2}{\lambda }-3)+4{C}_{5}(2\lambda +\frac{1}{{\lambda }^{2}}-3)(1-\frac{1}{{\lambda }^{3}}),\end{array}$$

where ${\sigma }_{3p}$ and ${\sigma }_{5p}$ are Cauchy stresses values and $\lambda =\frac{l}{{l}_{0}}=\varepsilon +1$ – stretch ratio (while *ε* is engineering strain).

Certain conditions on Mooney-Rivlin parameters are necessary^21^ to satisfy the stability criterion:

4 $$\frac{\partial {\sigma }_{ij}}{\partial {\varepsilon }_{ij}}\ge 0,\partial {\sigma }_{ij}\partial {\varepsilon }_{ij}\ge 0,$$

where $\partial {\sigma }_{ij}$ is the stress increment tensor associated with the strain increment tensor $\partial {\varepsilon }_{ij}$ through the constitutive relation. The conditions caused by this limitation are as follows: ${C}_{1}+{C}_{2}\ge 0$ and ${C}_{3}\ge 0$ for 3-parameter Mooney-Rivlin model, ${C}_{1}+{C}_{2}\ge 0$, ${C}_{4}\ge 0$, *C*_5_ < 0 and ${C}_{3}+{C}_{4}+{C}_{5}\ge 0$ for 5-parameter Mooney-Rivlin model. We analyzed the applicability of various hyperelastic models both ways: with and without this criterion.

For Yeoh model corresponding formulas are:

5 $$W=\sum _{\mathrm{3,}i=0}{C}_{i}{({I}_{i}-\mathrm{3)}}^{i},$$

and under the same assumptions, the Cauchy stress is represented by the formula:

6 $$\sigma =\frac{F}{{S}_{0}}=\mathrm{2(}\lambda -{\lambda }^{-2})({C}_{1}+2{C}_{2}({\lambda }^{2}+2{\lambda }^{-1}-\mathrm{3)).}$$

The last considered model was Neo-Hookean, for which in the same notation strain energy density function is:

7 $$W={C}_{1}({I}_{1}-3),$$

and the uniaxial stress is:

8 $$\sigma =2{C}_{1}(\lambda -\frac{1}{{\lambda }^{2}}),$$

where *C*_1_-linear part of elastic energy (Young modulus). We considered this most elementary hyperelastic model due to numerous papers^22^, where Neo-Hookean model is used for modelling the mechanics of cerebral aneurysm wall and solving the FSI problem.

At all cases the approximation of experimental data was carried out considering the non-negativity of linear coefficient in energy density formula. For Yeoh model coefficient *C*_1_ defines the linear component of energy, while both *C*_1_ and *C*_2_ in summation define the non-linear component. Mooney-Rivlin models are significantly more complicated: even linear energy component is defined by several coefficients. As for 3-parameter model it is *C*_1_ − 3*C*_3_ and for 5-parameter it is ${C}_{1}-3{C}_{3}-6{C}_{4}+4{C}_{5}$. That means Mooney-Rivlin models consider how the non-linear component affects linear component. When fitting the model curves to the experimental ones, we controlled the value of the coefficient in front of the linear term (we imposed the condition on the linear part that it cannot be negative), preserving the adequate physical nature of the hyperelastic model.

During the calculation the coefficients for various hyperelastic models describing a series of experimental data, we faced the problem of choosing an interval of strain ratio for describing the mechanical characteristics of a material using different hyperelastic models. For all patients, this interval was different both in terms of separate case and in terms of the loading stage (small deformations, average deformations, large deformations). We used the semi-robust technique to calculate the limiting value of the relative elongation (see Wolfram Mathematica module in the supplementary materials) for which the hyperelastic model approximates the experimental data with a given accuracy. To analyse the quality of fit we used value of normalised root mean square error (NRMSE), which is commonly used for such tasks^23^:

9 $$NRMSE=\frac{\sqrt{\frac{1}{n}\mathop{\sum }\limits_{i=1}^{n}{({y}_{ei}-{y}_{mi})}^{2}}}{|\frac{1}{n}\mathop{\sum }\limits_{i=1}^{n}{y}_{ei}|},$$

where n is the number of experiment data points, *y*_*ei*_ is experiment value and *y*_*mi*_ the value of the model.The essence of the error calculation method is the standard normal deviation.

## Results

As a result of experimental data processing Table 2 was created where coefficients of Yeoh, Neo-Hookean, and 3 and 5-parameter Mooney-Rivlin models are shown at the moment of maximum relative elongation, as it was done in^10,17^, while the approximation was still accurate enough.

**Table 2:** Values of coefficients for all models used in this work in the moment of ultimate stress.

ID	Mooney-Rivlin(3p) parameters, MPa			Yeoh parameters, MPa		Neo-Hookean parameter, MPa
	C1	C2	C3	C1	C2	C1
	Mooney-Rivlin(5p) parameters MPa					
	C1	C2	C3	C4	C5	
K2	0.464950	−0.464947	9.17e-7	0.021704	0.031802	0.0615
	0.46492	−0.464896	2.552e-6	2.334e-7	−1.598e-6	
R.	0.313226	−0.313226	1.863e-6	2.678e-7		0.032626
	0.12038	−0.12038	−0.040056	0.040069	−0.000011	
V.	0.479274	−0.43089	0.159734	4.77e-7	0.0379351	0.100453
	0.618282	−0.51001	−0.205882	0.205964	−0.000018	
Z	0.204286	0.070981	0.068063	1.12581	0.172556	0.123172
	0.2717	0.0274076	−0.089769	0.090082	−0.000059	
U.	0.085436	−0.07656	0.028474	7.85e-7	0.007087	0.116763
	0.075683	−0.034887	−0.022802	0.024003	−9.154e-6	
K1.	0.029655	0.021101	0.009883	1.07937e-6	0.007273	0.058434
	0.056429	0.005771	0.008226	0.000468	−0.00861	
M.	0.046188	−0.046162	0.015390	1.25635	0.095805	0.045382
	0.052108	−0.05119	−0.017356	0.01736	−1.652e-6	
Ul.	0.0263132	−0.00878	0.017	0.0002	0.000229	0.0099307
	0.021751	−0.004683	0.0005	0.000782	−0.000699	

**Figure 3:**
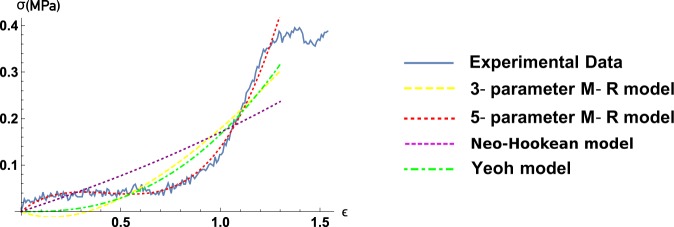
Typical strain-stress curve, 3 and 5 Mooney-Rivlin models, Neo-Hookean and Yeoh models approximations.

After analysis it turned out that Mooney-Rivlin models do not have any significant advantages over Yeoh model for small strain values. However as strain value increases, Yeoh model stops to approximate experimental data sufficiently. As it is shown on Fig. 3 even 3-parameter Mooney-Rivlin model does not approximate with satisfying accuracy, with little relation to usage of stability criterion. Only 5-parameter Mooney-Rivlin model adequately approximates experimental data for both small as well large strain values.

Complex models, such as ones described in^24^, have much wider range of usage, however they start to approximate the experimental data well enough only starting with quite big values of relative elongation ($\lambda \ge 1.76$)^24^. The analysis of the results on the limits of hyperelastic models applicability summarised on the Figs 3 and 3 show a good applicability of hyperelastic models both at the stages when the elastin matrix carries the main tension load, and at the beginning of that zone when only collagen fibers bear the loading.

**Figure 4:**
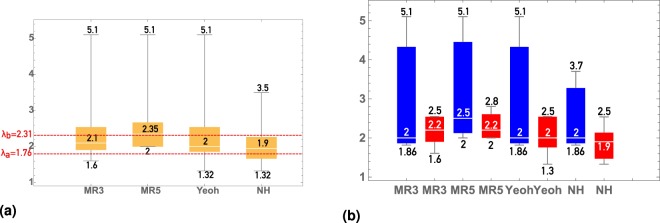
Distribution of values of maximum strain, with the approximation remaining sufficient. The stress-strain curves up to those values are approximated by models with sufficient quality. By vertical axis – distribution of maximal strain values, the limits of the applicability of hyperelastic models. The displayed values stand for (from high to low) upper fence, 75% quantile, mean, 25% quantile, lower fence. MR3 corresponds to Mooney-Rivlin 3 parameters model, MR5 corresponds to Mooney-Rivlin 5 parameters model. Meaning of *λ*_*a*_ = 1.76 and *λ*_*b*_ = 2.31 is explained in^24^. Fig (**b**) compared to (**a**) shows the importance of accounting status (ruptured/unruptured) of IA.

Figure 4a shows that the zone of large deformations (*λ* > *λ*_*b*_) when in the fibrous structure of the sample is mainly involved in the loading) captures only the MR5 model, whereas at small deformations (*λ* < *λ*_*a*_) the NeoHookean model shows good applicability to both ruptured and unruptured aneurysms. In the interval (*λ*_*a*_ < *λ* < *λ*_*b*_) we observe different mean values, which are shown by the NeoHookean and Yeoh models for ruptured and unruptured aneurysms, respectively. The result is especially interesting because the struggle of choosing the best model happens around the interval (*λ*_*a*_ < *λ* < *λ*_*b*_), which characterizes the involvement in the mechanical work of the elastic matrix, fiber or both, as shown in^25^.

We have also carried out the comparison for ultimate stress and strain (see Table 3) for two cohorts of experimental samples (ruptured and unruptured), as it was done in^17^. The Mann-Whitney test for small samples was chosen and showed no statistically significant difference for ultimate strain was discovered for specimens from our research even for $p\le 0.05$, while there was shown statistical significance for differences in values of ultimate stress for $p\le 0.01$.

**Table 3:** Maximum strain ratio values.

ID	Maximum relative elongation			
	Mooney-Rivlin (3p)	Mooney-Rivlin (5p)	Yeoh	Neo-Hookean
K1	5.10488	5.10488	5.10488	3.70363
K2	1.81725	1.81725	1.81725	1.81725
R.	2.00078	2.00078	2.00078	2.00078
V.	1.60622	2.003	1.32909	1.32909
Z.	2.00094	2.00094	2.00094	2.00094
U.	2.54697	2.80683	2.54696	1.5161
M	0.2.20088	2.20088	1.90279	1.90279
U1.	2.53922	2.53922	2.53922	2.53922

It turned out that various hyperelastic models describe experimental data for ruptured and unruptured aneurysms at completely different *λ* intervals. Ruptured aneurysms are characterized by good approximation of the considered models in the zone of small and medium deformations (see Fig. 4b), at the same time for unruptured aneurysms the considered models work for small, medium, and large deformations. Basing on the obtained histograms on Fig. 4b we created the Table 4 of recommendations on choosing the hyperelastic model depending on the particular task.

**Table 4:** Optimal hyperelastic model of aneurysm tissue, based on the status of the aneurysm and the level of its deformation. Small deformations correspond to *λ* ≤ *λ*_*a*_, medium deformations correspond to *λ*_*a*_ ≤ *λ* ≤ *λ*_*b*_, large deformations correspond to *λ*_*b*_ ≤ *λ*.

Rate of deformation/Aneurysm status	Ruptured	Unruptured
Small deformations	Neo-Hookean	Neo-Hookean
Medium deformations	Yeoh	Neo-Hookean model
Large deformations	Mooney-Rivlin 5 parameter model	Mooney-Rivlin 5 parameter model

## Discussion

It is worth noting a few limitations that were present during this work. First limitation was performing the test in air, rather than in a thermostatic bath, as such tests were conducted in^10,16,17^. However, we are convinced that such study is relevant for a number of reasons. First, in modern neurosurgical practices there are many methods in which a blood vessel remains “dry” (not filled with blood, and in the absence of perivascular space substance) for a long period of time (up to several hours) while being periodically moistened with saline, and fully restores its physiological properties after the resumption of blood flow and perivascular environment. Second, paper^20^ presents the results of comparing the experimental data of mechanical tests with specimens in the air and the saline, which do not show a significant difference. Therefore, conducting an experiment in such setup plays an important role. Restoration of mechanical properties was not described in the literature, and in the course of performing the experiments described in our work, we resorted to wetting the sample located in the clamps with saline (0.9%) using a spray bottle. Besides, as the practice of such studies shows^16,17^, the mechanical properties of samples of intracranial aneurysms have a very high diversity depending on the conditions of the experiment, and it only makes sense to compare different sets of mechanical properties of intracranial aneurysms, ruptured and unruptured, measured under the same conditions, as it was done in this work.

Another aspect limiting this study is the uniaxial approach to the mechanical experiment, as the walls of the vessels are essentially anisotropic material even in the case of healthy vessels, not to mention a cerebral aneurysm which wall has more complex structure^19^. We considered hyperelastic models in the work on the assumption that the material of the wall of the cerebral aneurysm is homogeneous, which is more natural for constructing the most common grids in the literature for modelling material, since assuming the structure of the material is fibrous, it would be more correct in this case to consider the hierarchy of models of GOH type (generalized Ogden-Holzapfel models), which is partly done in^25^. An obvious solution to this problem is a biaxial mechanical experiment. However, as many studies show, there are many problems when performing an experiment in this case: from choosing the shape of the test sample to choosing the method of fixing the material and analysing the deformation zone. In particular, it was shown that the zone of two-dimensional deformations with a biaxial test is extremely small, which, together with the extremely small size of tissue samples of the wall of cerebral aneurysms, makes conducting such an experiment difficult. The semi-biaxial test is known^15^, however, a significant limitation with respect to this type of research is the required size of the sample (the sample must be wide enough), which is almost impossible to achieve with intracranial aneurysms.

The software of the machine we used did not allow to measure the relaxation of material during its restoration to original state after each measurement. A series of experiments with consistent increase in the engineering strains of the material in the course of the experiment is, in fact, analogous to the natural growth of aneurysm and thinning of its wall. The purpose of such experiments is to assess the changes in the mechanical characteristics of an aneurysm with its growth (Fig. 2).

In the course of conducting experiments and analysing the obtained results, we were convinced that the obtained experimental results in general correspond to the results already described in the literature. Of course, the results obtained (strain-stress relationships, Neo-Hookean, Yeoh, and Mooney-Rivlin parameters) differ from those given in the literature^10,17,20^, however, as mentioned above, for example, in^17^, the significant factor in comparing the results is the conditions of experiment: used tensile testing machine, method for determining the deformation of the material, control of temperature and humidity of the material, method of conservation before experiment. In particular, in^25^, the advantages of choosing the dog-bone shaped type of specimen to achieve its rupture in the centre (in length) rather than near the clamps are shown. However, this form of material has several drawbacks: it is necessary to take into account that even when considering hyperelastic models of sample mechanics, in fact, the test material is fibrous and similar excision leads to an uneven distribution of fiber families (with angle to the central axis) in the sample along its length, which makes it impossible to correctly process the results of an experiment only when controlling the deformation. However, it is worth noting that this technique is good for observing the rupture front in the sample, since the point of rupture is determined by sample’s shape. In our case the measured geometric characteristics of the sample which allow us to calculate stresses correctly, have more important role. The choice of the direction of deformation relative to the main direction of collagen fibers in the sample may be another important factor. Collagen fibers, as shown in the literature, have a Poisson distribution and preserve that distribution under moderate uniaxial tension^26^.

In the work^16^ one can observe the punctures in the sample, which are necessary to control the change in true strain value. In our protocol, since it was not possible to capture the measurements visually, we minimised the distance between clamps to lower the effects of edge disturbance.

The methods currently used, like punctures of a sample, also have their limitations, namely: the puncture point is prone to unpredictable deformation, which does not allow us to unambiguously judge the correct tracking of deformation markers. To eliminate the limitations associated with the methods used in the work, one should more accurately take into account the deformation of the sample. Namely, it is proposed to develop a technique for gluing markers and tracking their movement, as is done, for example, in^27^. A similar approach will allow to obtain true uniaxial deformation of samples with minimal impact of edge effects.

The measurement of the change in the shape of the sample (cross-sectional area in various places) after the test was not carried out, since we have not seen such data anywhere in the reviewed literature. However, we believe that the assessment of such changes will allow verification with the results of maximum wall displacements and stresses on it by FSI calculations. Such a measurement could be carried out using a notched substrate for an aneurysm sample and using 2 video extensometers located on the sides of the sample during a mechanical test. Thus, we could determine not only the change in the width of the sample, but also the change in its thickness (although only near the edges).

Another limitation of this study is not considering the patients medical records and factors that may affect the tissue properties: systemic diseases, smoking, other diseases listed in Table 1. In the literature there are many works^28,29^ describing the influence of systemic and hereditary factors that influence the risk of IA rupture, however in this work our attention is focused on mechanical properties of tissue and hyperelastic models, and describing the experiment. We considered that obtaining statistically significant differences in the ultimate stress of specimens of ruptured and unruptured aneurysms (which is an evident criterion, if we rely on the literature) will allow us to consider the number of collected specimens sufficient to perform the presented work.

In the future we plan to pay attention to the plateau area and the area of the strain-stress curve from the moment of the visible gap in aneurysm tissue. This area was not analysed in this work and we did not find relevant studies in the literature. At the same time this part of strain-stress diagram corresponds with the moment of rupture of the tissue of the aneurysm. Mechanics of a such stage of aneurysm rupture process is currently not elucidated and is of great interest for the development of the fundamentals of the mechanics of vascular tissues.

We wish to note the wide applicability of the results we obtained, allowing to select an optimal hyperelastic model for cerebral aneurysm wall for every specific FSI hemodynamics task, while following the physics of the modelled process.

## Conclusions

In the course of this study, mechanical tests were performed on eight cerebral aneurysms. The range of applicability of hyperelastic models for modeling cerebral aneurysm tissues depending on the achieved relative elongation is determined for the first time, taking into account the physical adequacy of the use of models of hyperelastic material. The obtained result lets other researches choose an hyperelastic model for aneurysm tissue modelling in accordance with their specific task of numerical hydroelastic modelling of hemodynamics of cerebral aneurysms.

### Additional Data

In this section, we provide additional information that may be useful.

**Figure 5:**
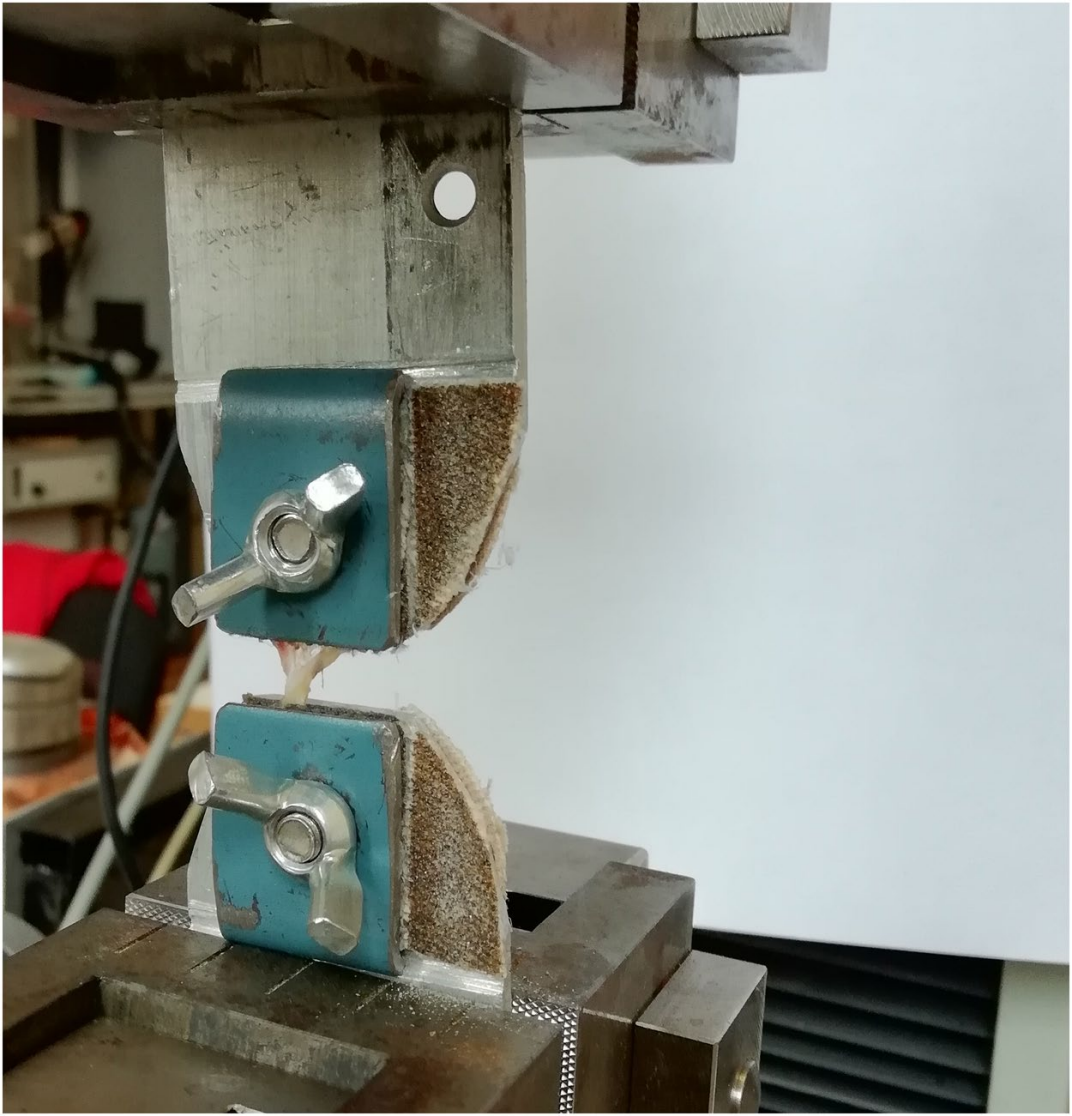
Specimen in tensile machine.

The specimen is mounted in a tensile testing machine against a contrasting background to more accurately determine the presence of visible ruptures in it.≫. (Fig. 5).

In addition, we wished to carry out the comparison of such between aneurysm walls and walls of healthy arteries. The value of such a comparison is hard to underestimate since the extraction of a healthy artery during neurosurgery is a rare occasion, unlike the extraction of aneurysm wall. In this section we present strain-stress diagrams of experiments with uniaxial loading of healthy temporal artery. The data of the patient, whose part of temporal artery was removed during the ablative brain surgery is stated in Table 1.

**Figure 6:**
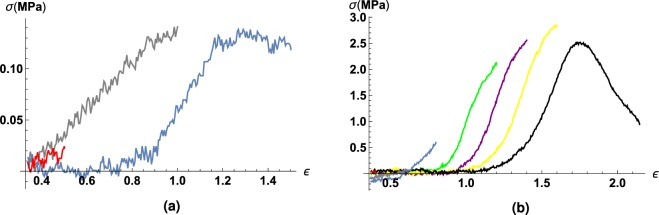
Strain-stress dependencies for case A. (healthy artery): (**a**) shows aforementioned relationship for tensile test circumferentially, (**b**) - axially.

As can be seen from Fig. 6 The values of the ultimate stress and strain for the axial and circumferential direction of deformation for healthy artery differ dramatically.

As mentioned, this work is bridging the gap in the area of knowledge about limits of hyperelastic models applicability to cerebral vessels tissues. And if in^25^ a selection of more or less complex models for a healthy artery was demonstrated, this work focuses precisely on the tissues of cerebral aneurysms.

The experiment with a healthy artery showed a dramatic difference in the values of the ultimate stress and strain in tension of two adjacent parts of the artery in axial and circumferential direction. This suggests the importance of controlling the main direction of the distribution of fibers in the material during the experiment. The calculated constants of hyperelastic models were substantially different in comparison with the case of both ruptured and unruptured aneurysms.

### Method of measuring geometric characteristics

**Figure 7:**
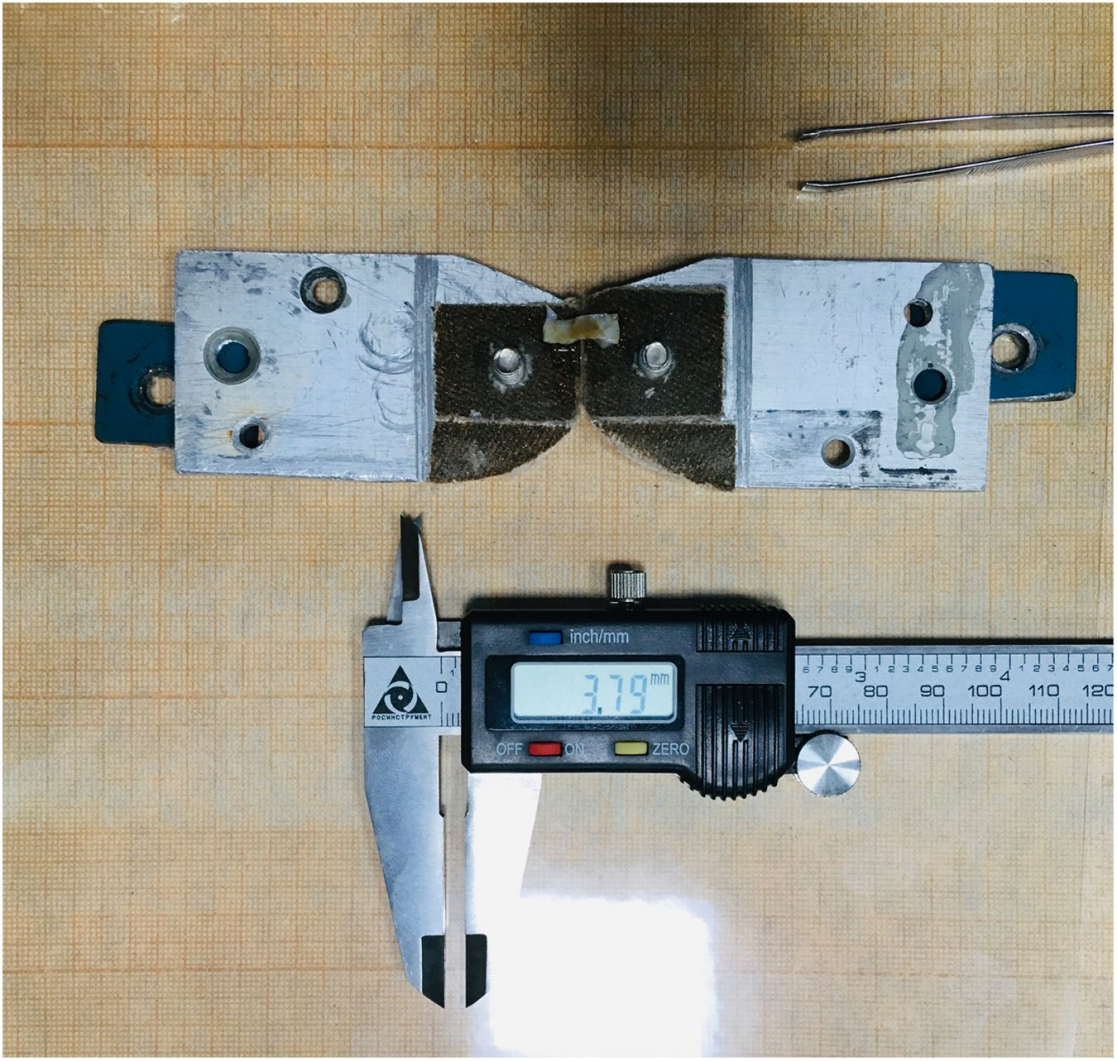
Measuring the characteristics of specimen of patient V.

As shown in Fig. 7, the sample, while measuring its geometric characteristics, is lying freely on the clamps, which in turn are located on the engineering paper and the laminated substrate. In the case when there was insufficient natural light, the measurement zone was illuminated with an LED lamp. The sample was measured mechanically using an electronic caliper, as well as visually (relatively), against a background of engineering paper, the data of both measurements were averaged. The thickness of the sample was measured using an electronic caliper with a decrease in the gap between the measuring girths until the condition for the onset of deformation of the sample was reached.

## Data Availability

All initial data on the force, absolute elongation of the samples, as well as the geometric characteristics of the samples are available for public download.
